# Supplementary figures and images for: Kap-β2/Transportin mediates β-catenin nuclear transport in Wnt signaling
Source: eLife. 2022 Oct 27;11:e70495. doi: 10.7554/eLife.70495 (PMC9665845; doi:10.7554/eLife.70495)

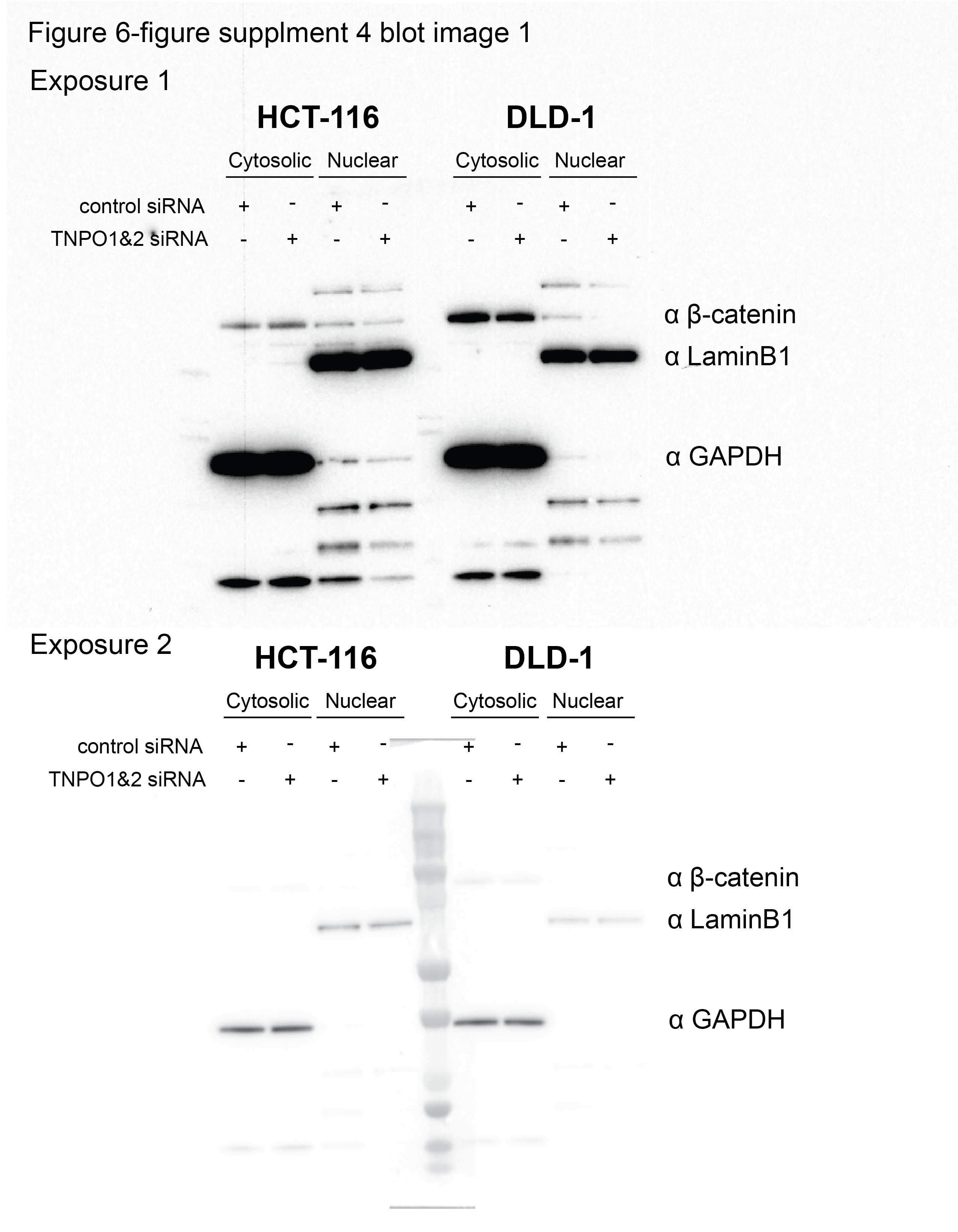

Supplement: Source data 1. [file elife-70495-data1.zip › Source data_gel & blot_revision_10_19_22/Figure 6-figure supplment 4 blot image 1.png]

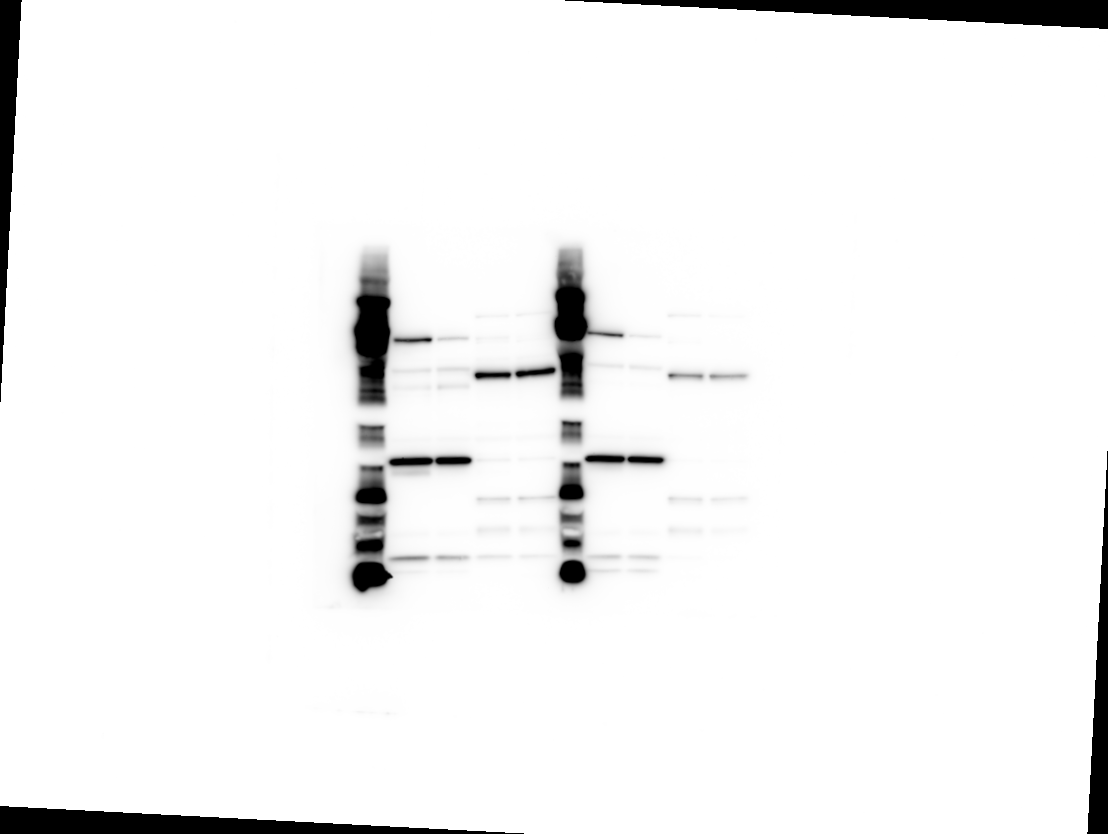

Supplement: Source data 1. [file elife-70495-data1.zip › Source data_gel & blot_revision_10_19_22/Figure 6-figure supplement 4 blot image 2 (bottom)_raw.tif]

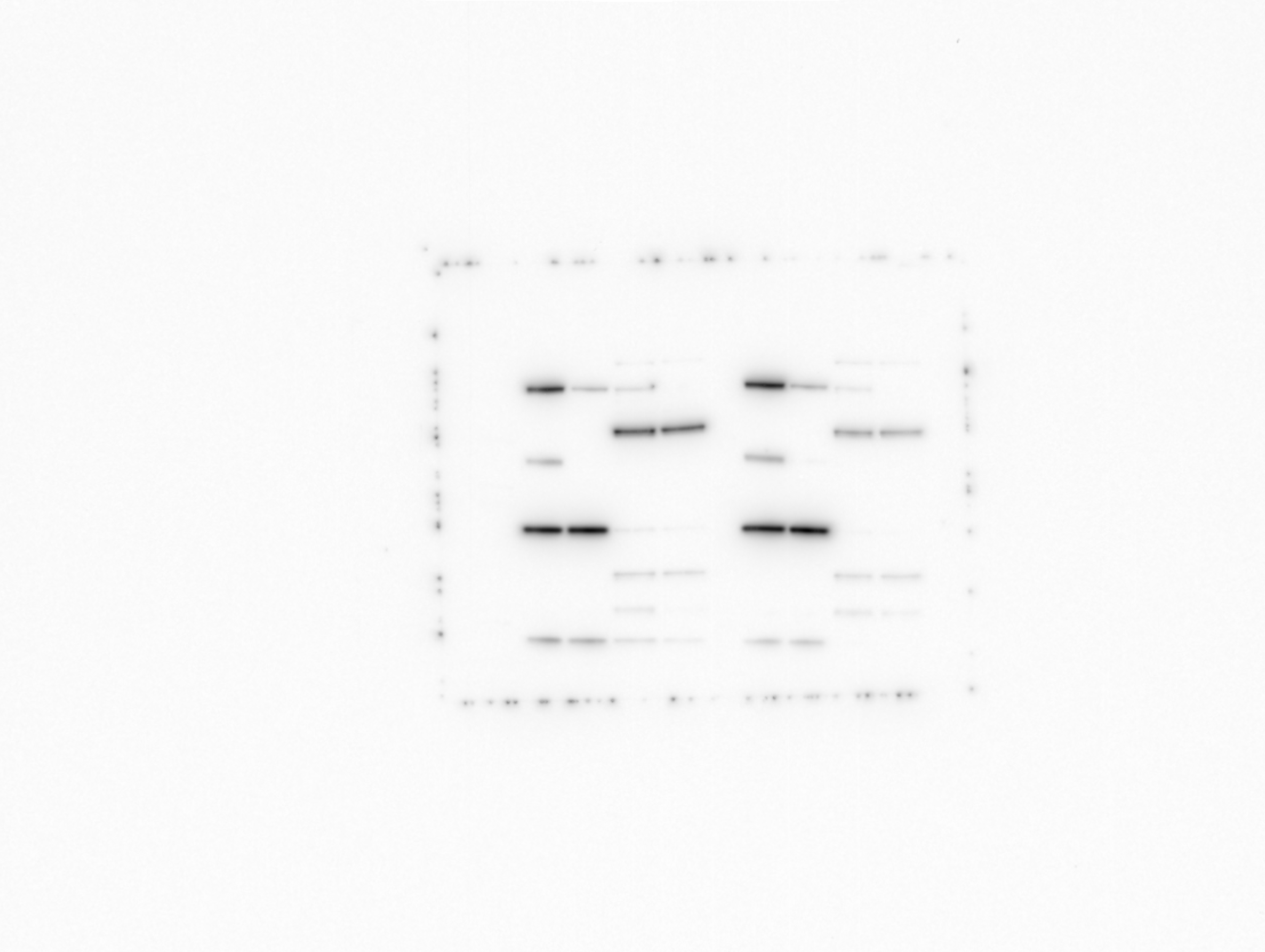

Supplement: Source data 1. [file elife-70495-data1.zip › Source data_gel & blot_revision_10_19_22/Figure 6-figure supplement 4 blot image 2 (top)_raw.tif]

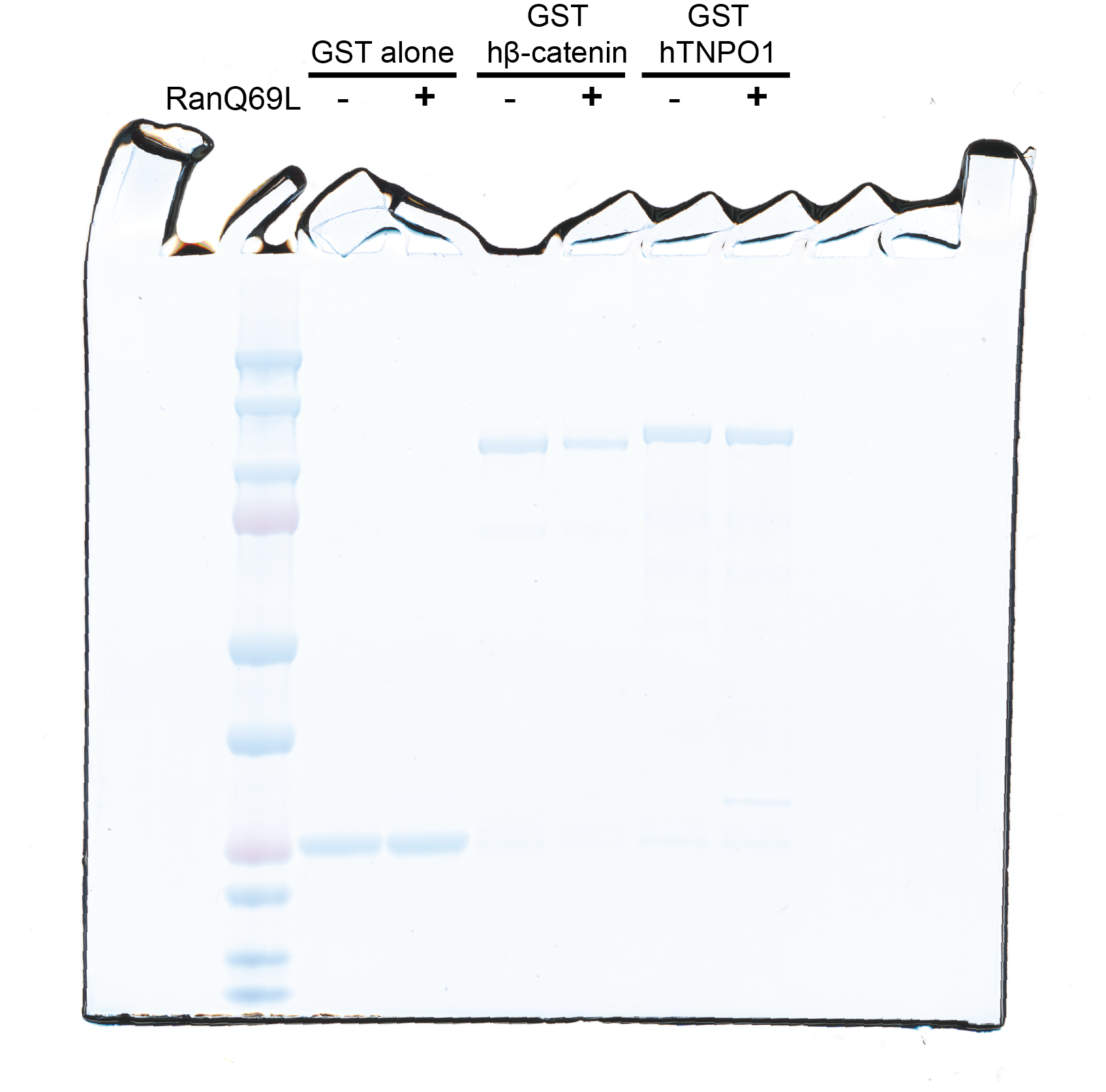

Supplement: Source data 1. [file elife-70495-data1.zip › Source data_gel & blot_revision_10_19_22/Figure 5-figure supplement 1B image.png]

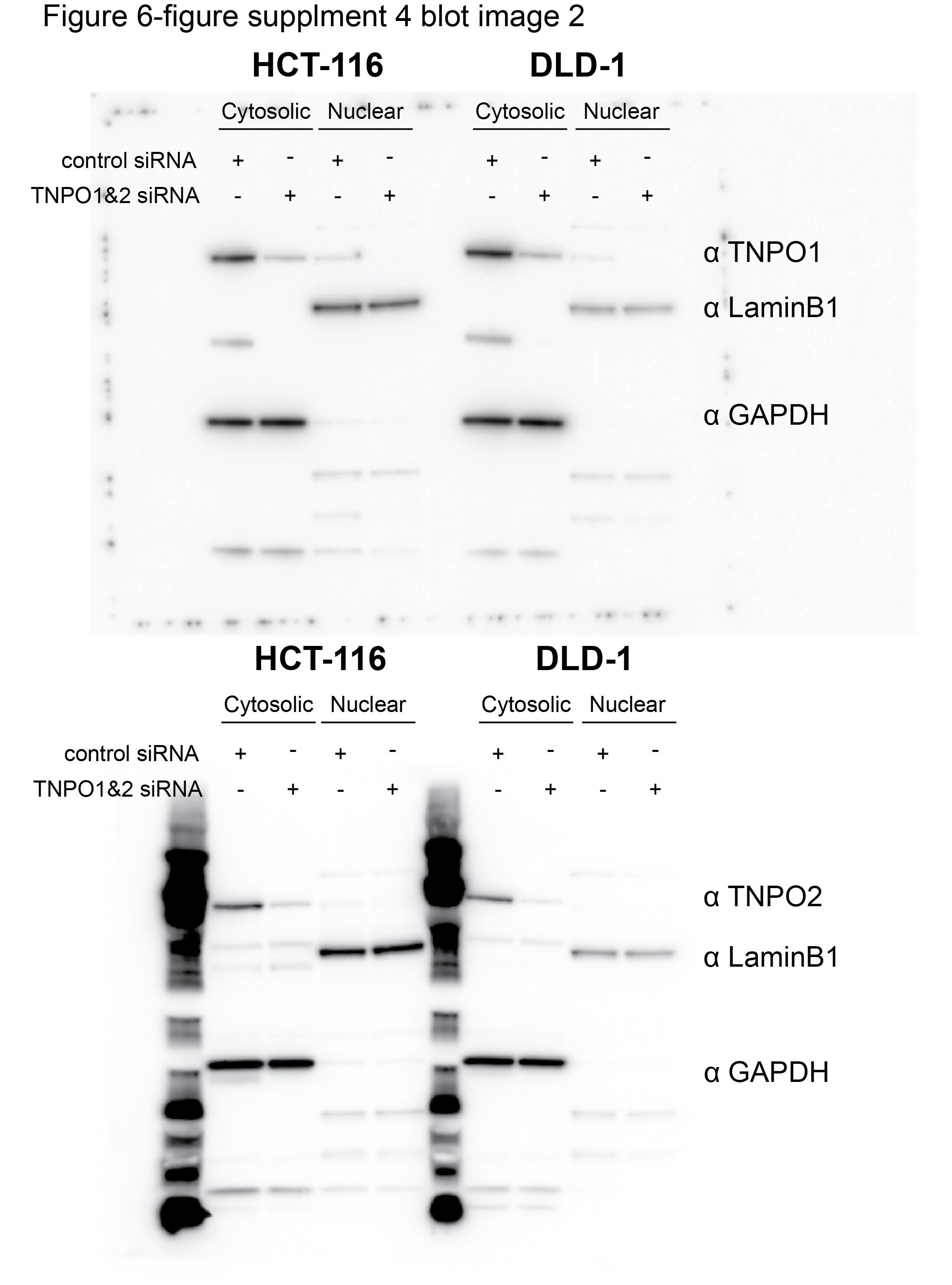

Supplement: Source data 1. [file elife-70495-data1.zip › Source data_gel & blot_revision_10_19_22/Figure 6-figure supplment 4 blot image 2.png]

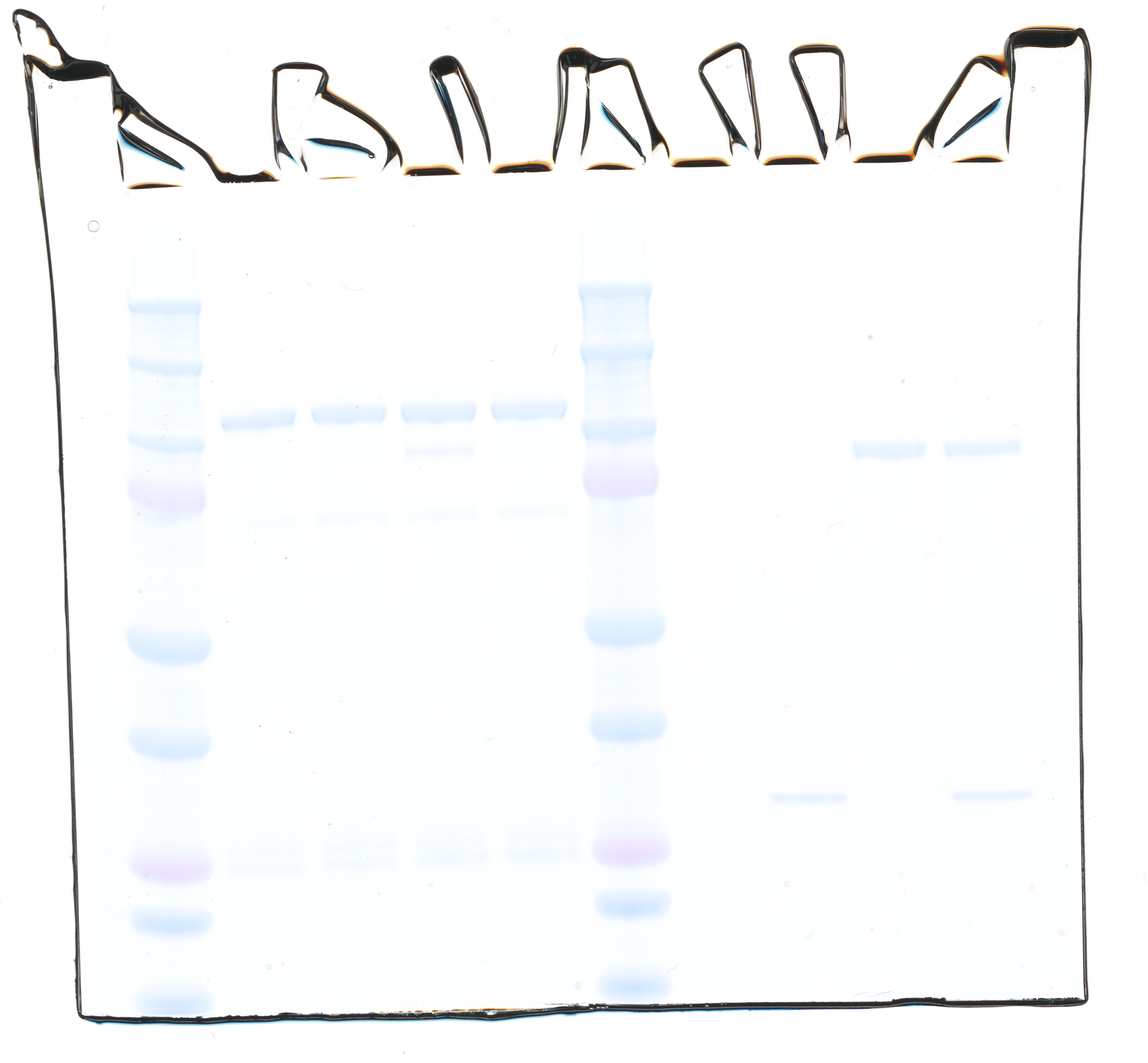

Supplement: Source data 1. [file elife-70495-data1.zip › Source data_gel & blot_revision_10_19_22/Figure 5B_raw.tif]

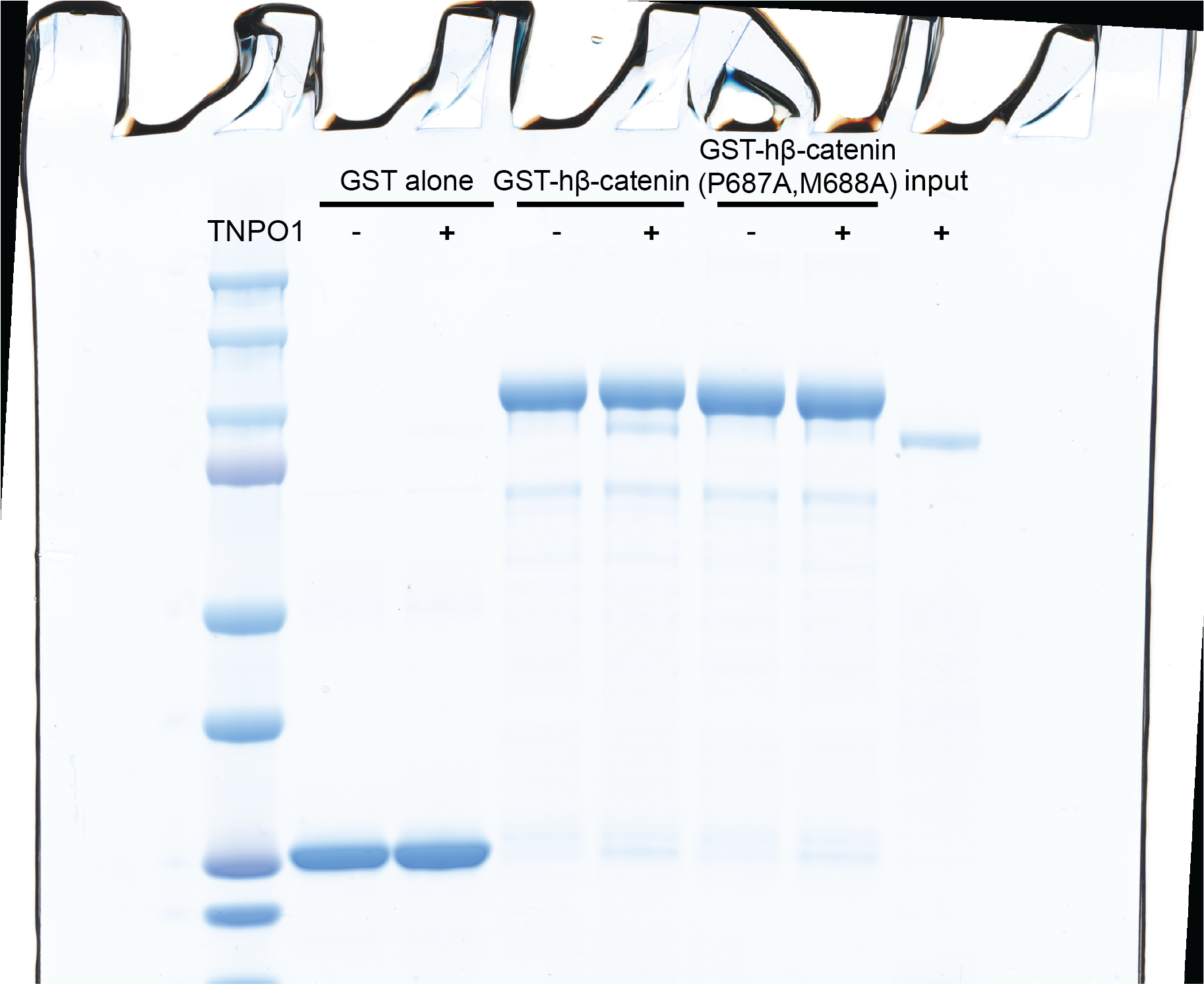

Supplement: Source data 1. [file elife-70495-data1.zip › Source data_gel & blot_revision_10_19_22/Figure 5A image.png]

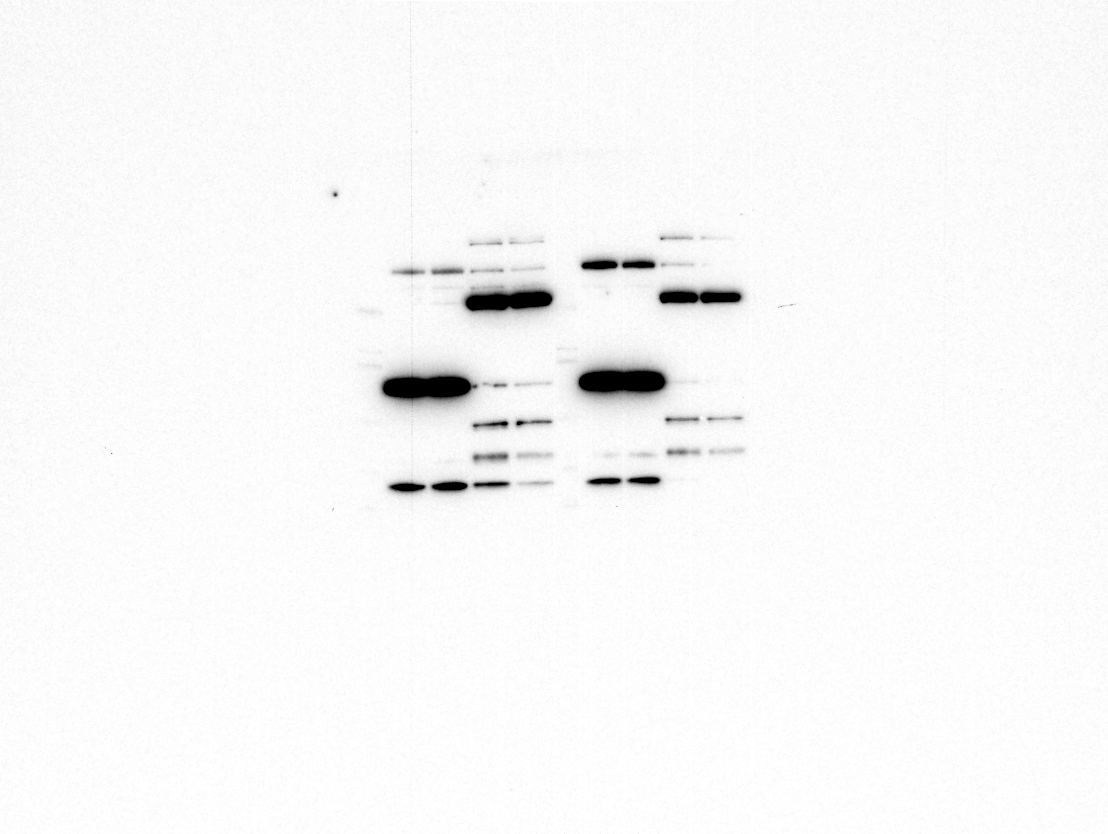

Supplement: Source data 1. [file elife-70495-data1.zip › Source data_gel & blot_revision_10_19_22/Figure 6-figure supplement 4 blot image 1 (top)_raw.tif]

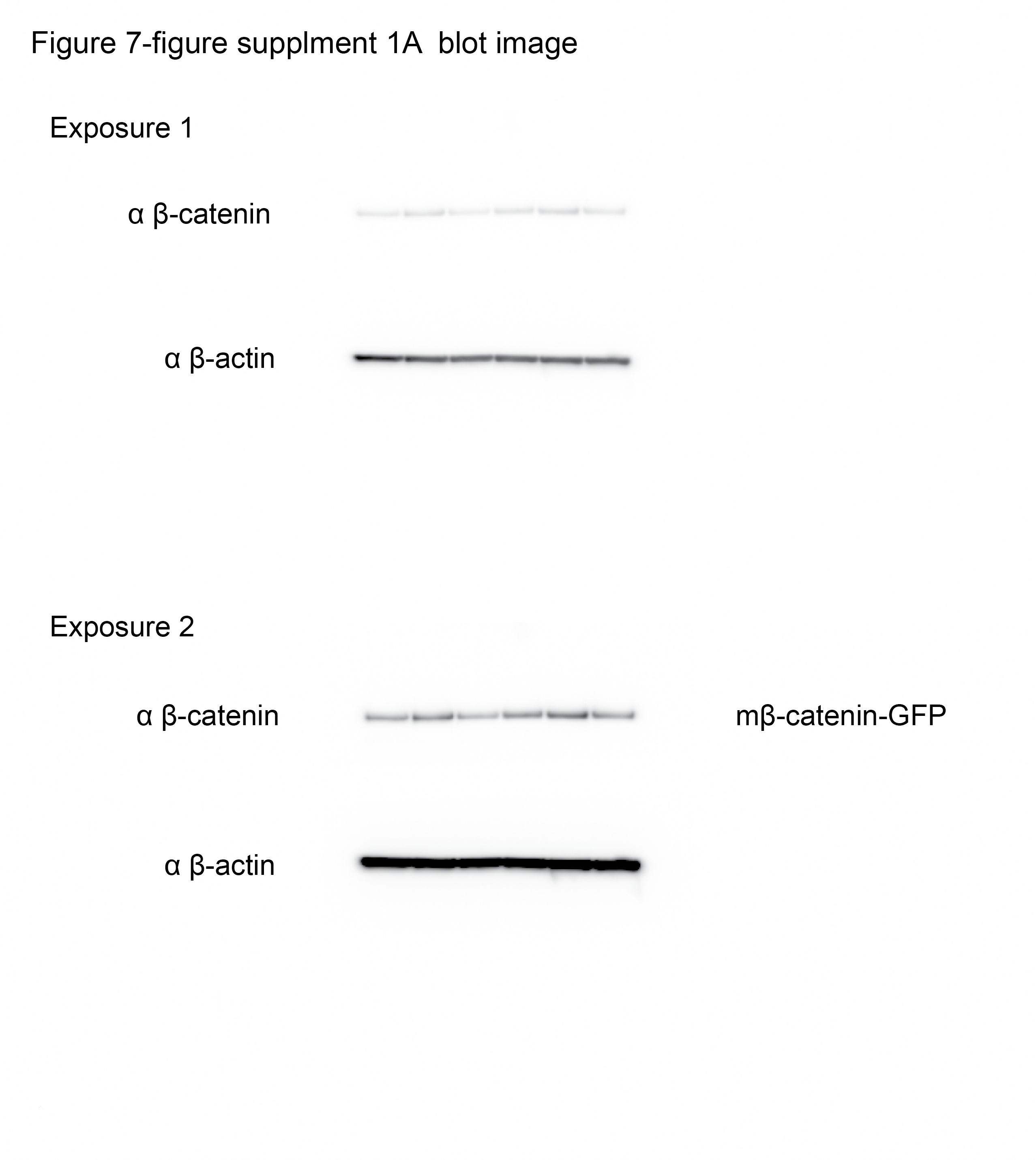

Supplement: Source data 1. [file elife-70495-data1.zip › Source data_gel & blot_revision_10_19_22/Figure 7-figure supplement 1A blot image.png]

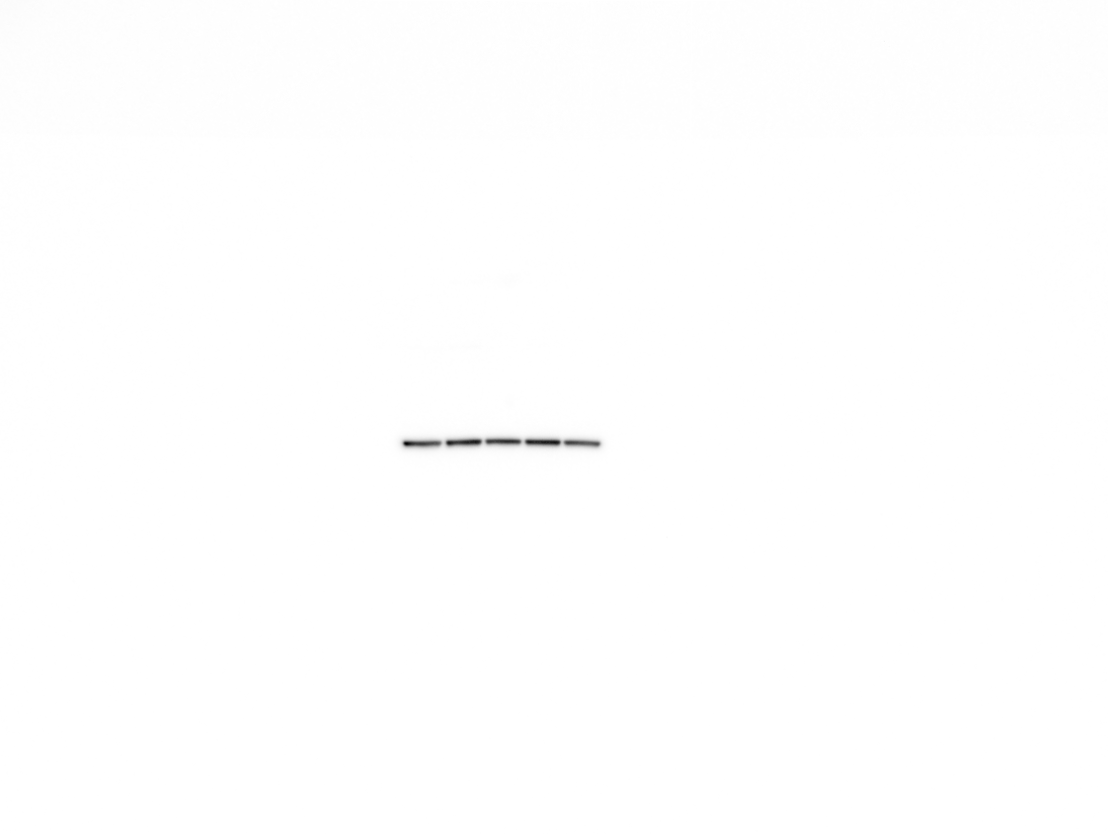

Supplement: Source data 1. [file elife-70495-data1.zip › Source data_gel & blot_revision_10_19_22/Figure 6-figure supplement 3 tnpo1 and b-actin blot (top left)_raw_exposure 1.tif]

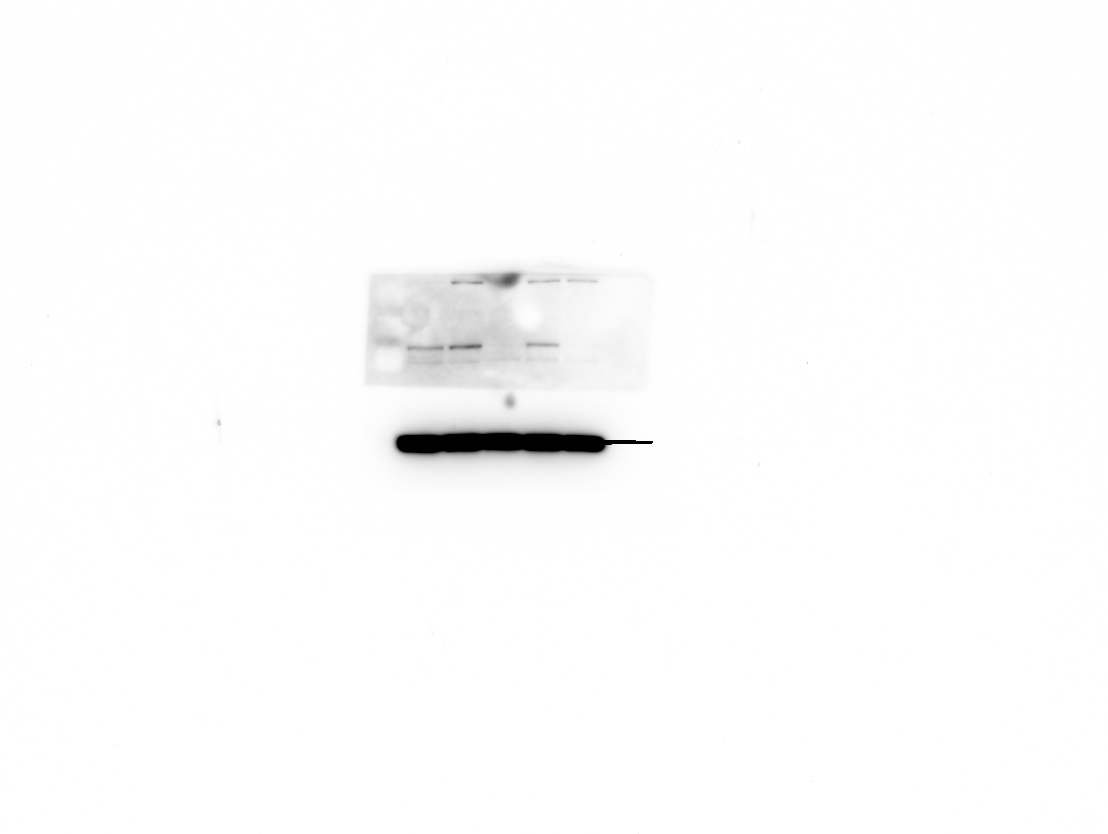

Supplement: Source data 1. [file elife-70495-data1.zip › Source data_gel & blot_revision_10_19_22/Figure 6-figure supplement 3 tnpo1 and b-actin blot (top left)_raw_exposure 2.tif]

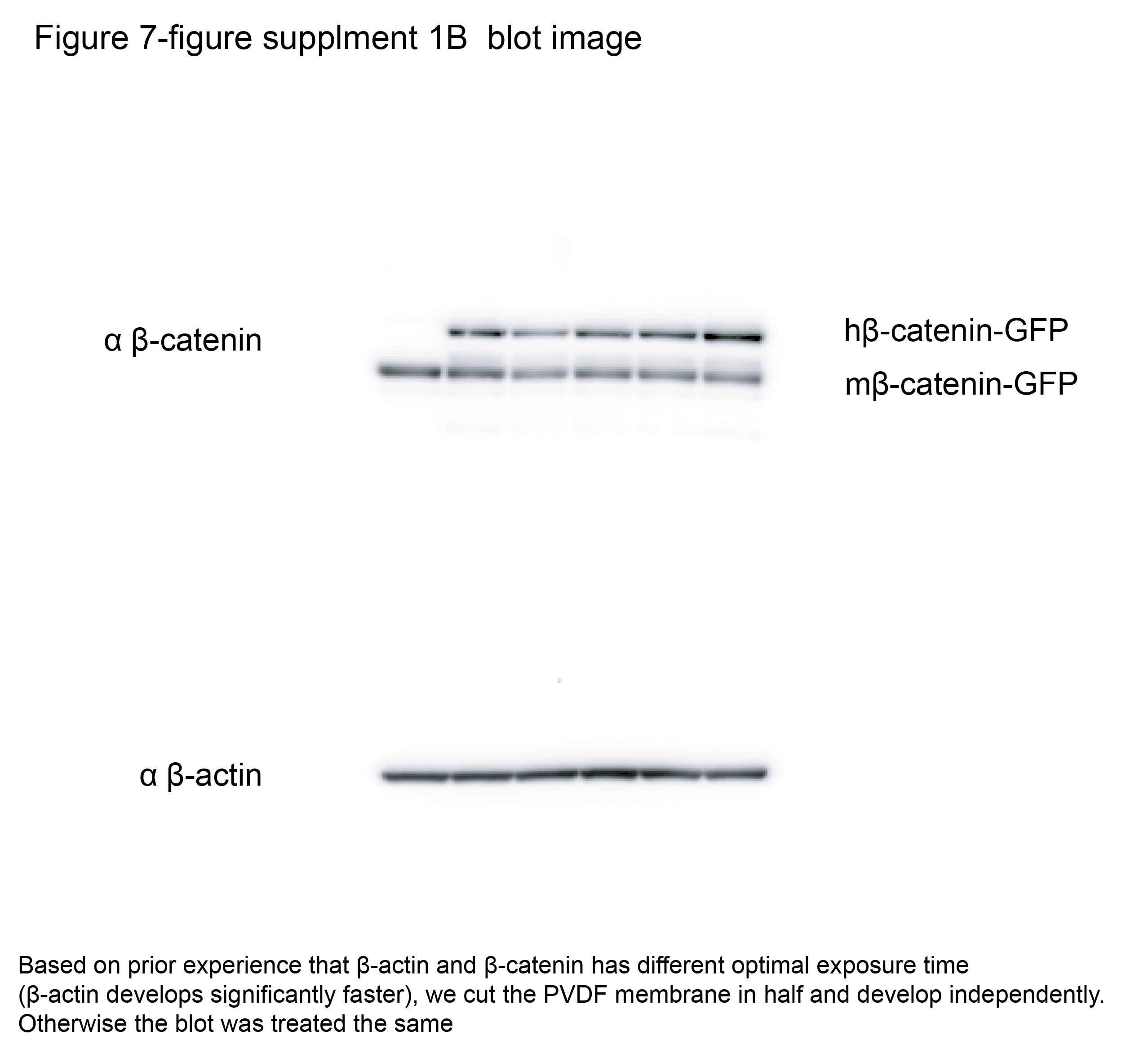

Supplement: Source data 1. [file elife-70495-data1.zip › Source data_gel & blot_revision_10_19_22/Figure 7-figure supplement 1B blot image.png]

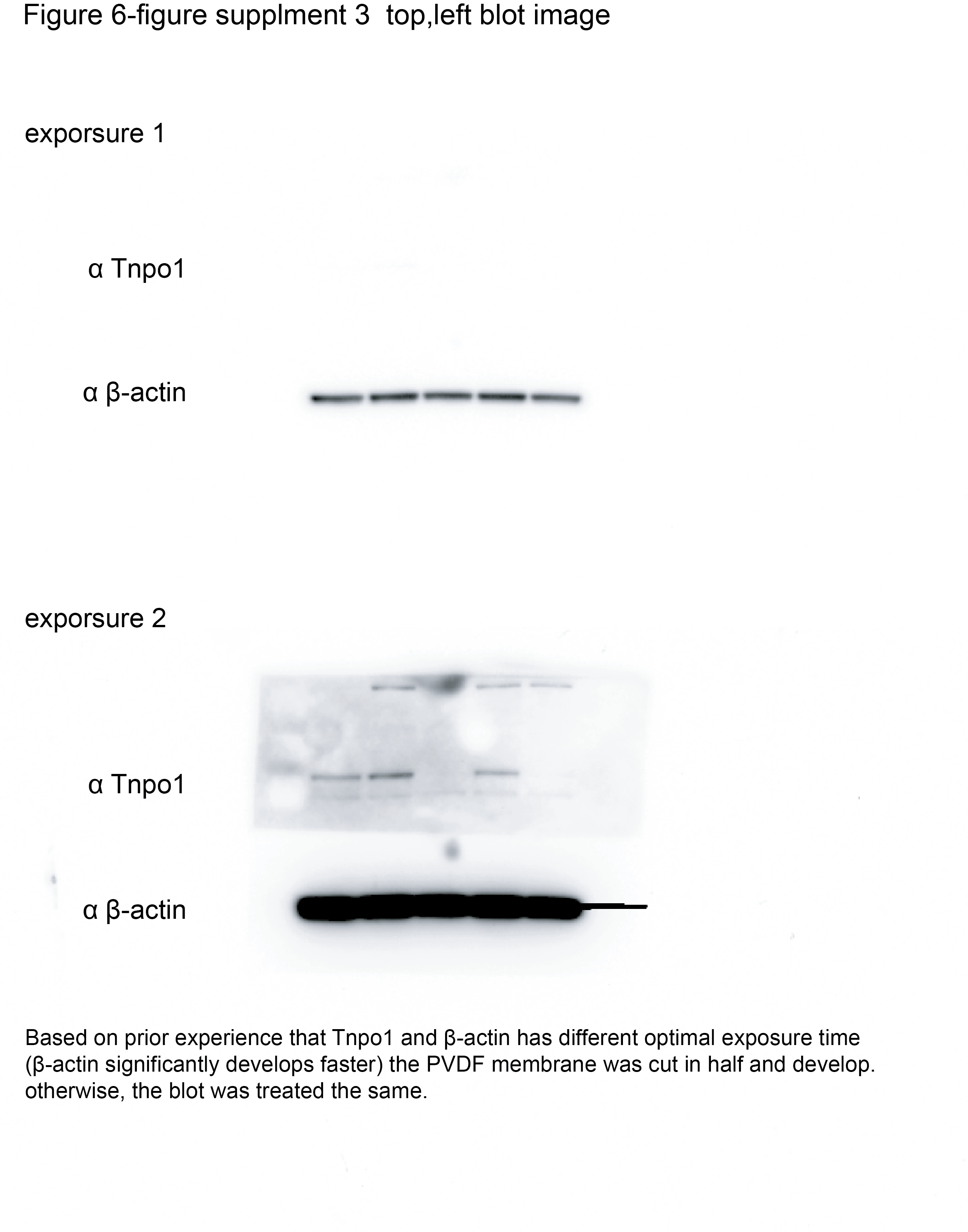

Supplement: Source data 1. [file elife-70495-data1.zip › Source data_gel & blot_revision_10_19_22/Figure 6-figure supplement 3 top left blot image.png]

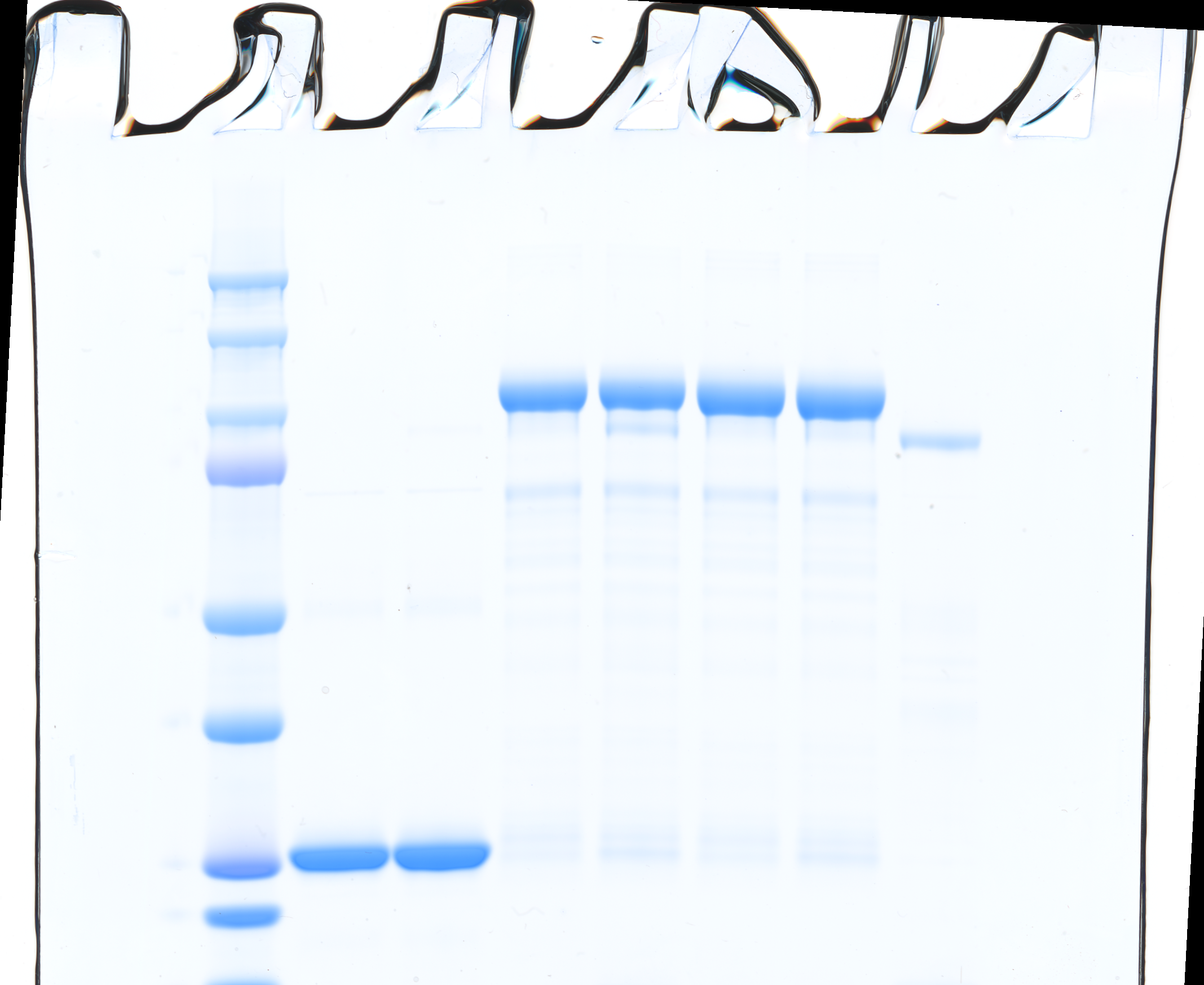

Supplement: Source data 1. [file elife-70495-data1.zip › Source data_gel & blot_revision_10_19_22/Figure 5A_raw.tif]

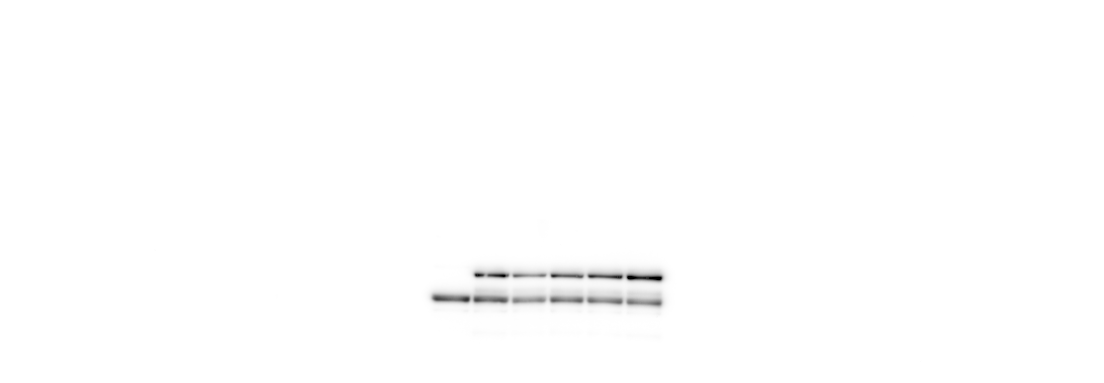

Supplement: Source data 1. [file elife-70495-data1.zip › Source data_gel & blot_revision_10_19_22/Figure 7-figure supplement 1B b-catenin ab_raw tif .tif]

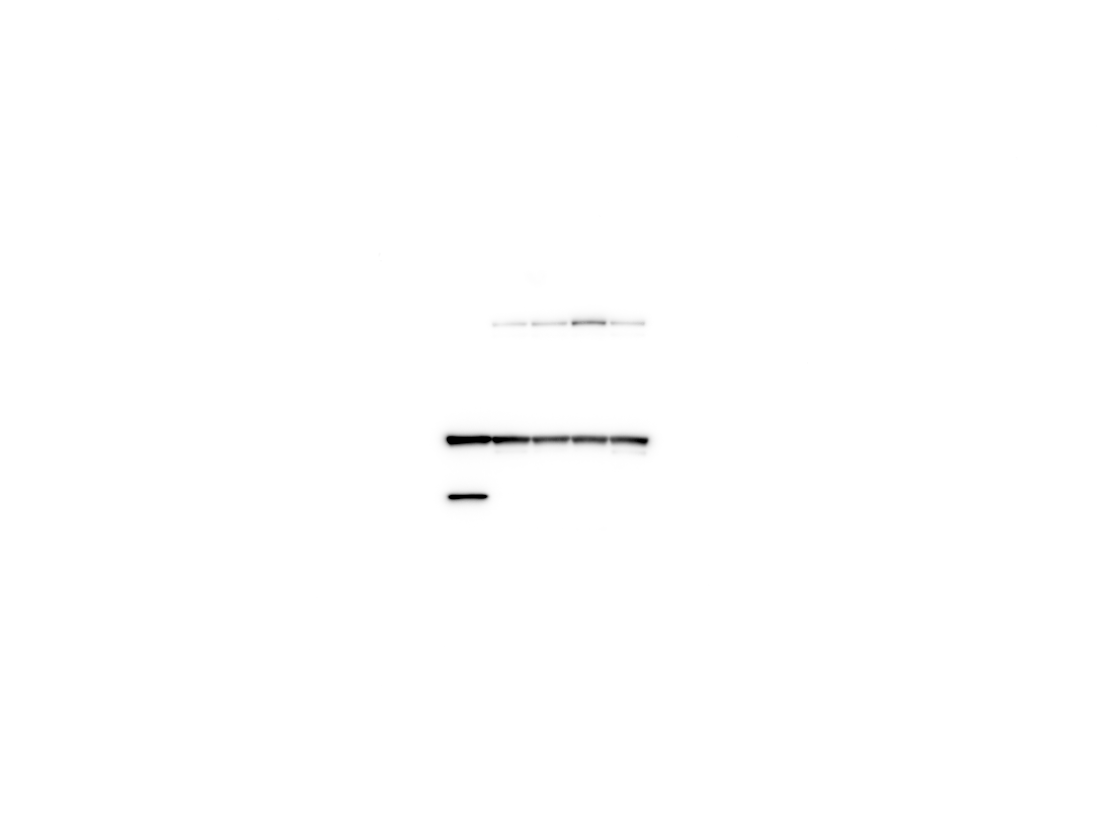

Supplement: Source data 1. [file elife-70495-data1.zip › Source data_gel & blot_revision_10_19_22/Figure 6-figure supplement 3 GFP and b-actin blot (bottom right)_raw.tif]

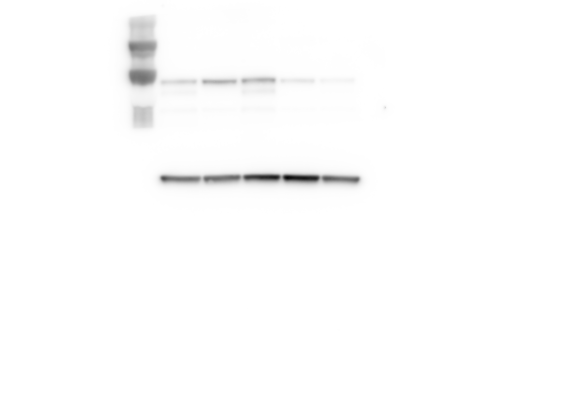

Supplement: Source data 1. [file elife-70495-data1.zip › Source data_gel & blot_revision_10_19_22/Figure 6-figure supplement 3 tnpo2 and b-actin blot (top right)_raw.tif]

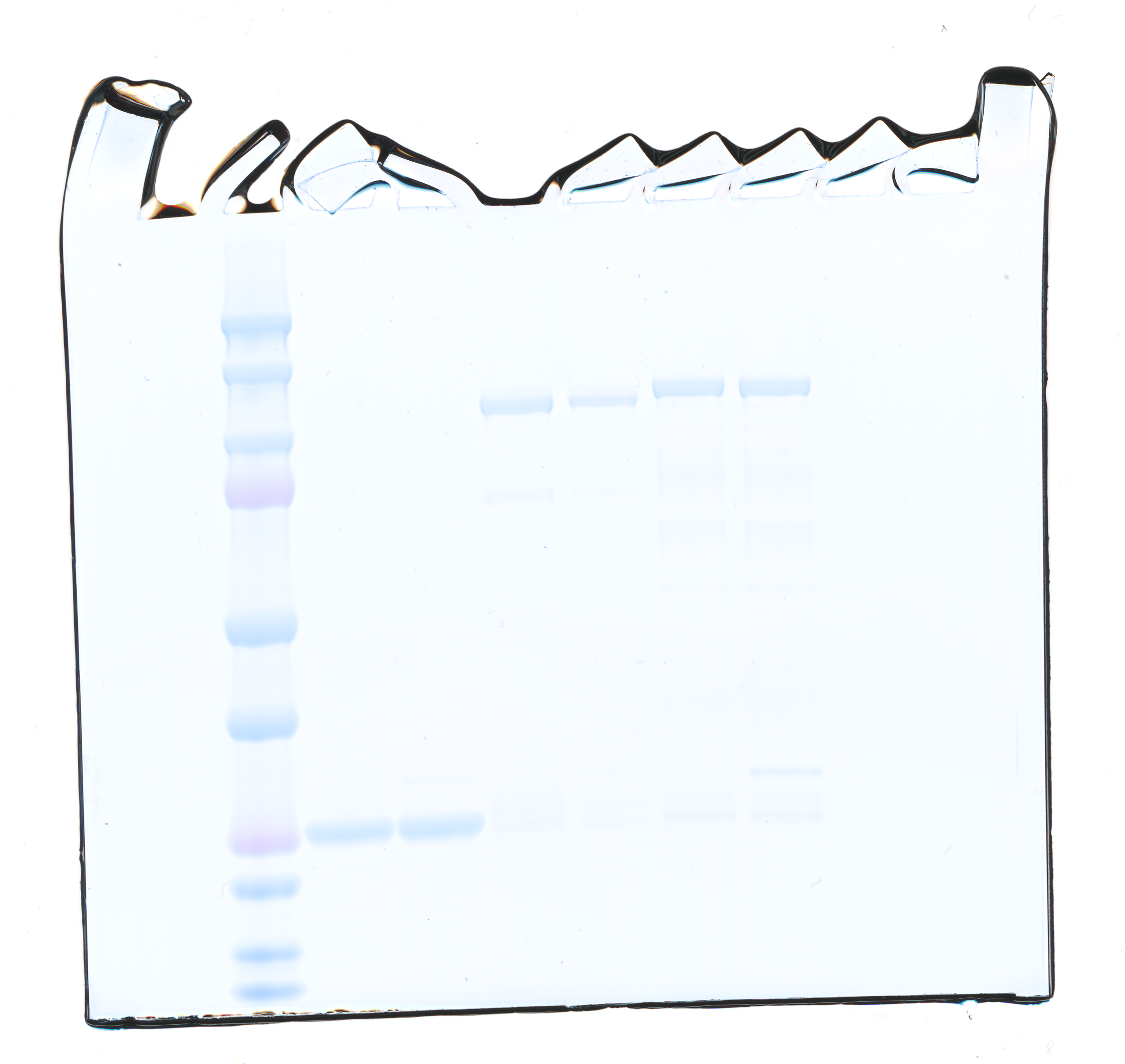

Supplement: Source data 1. [file elife-70495-data1.zip › Source data_gel & blot_revision_10_19_22/Figure 5-figure supplement 1B_raw.tif]

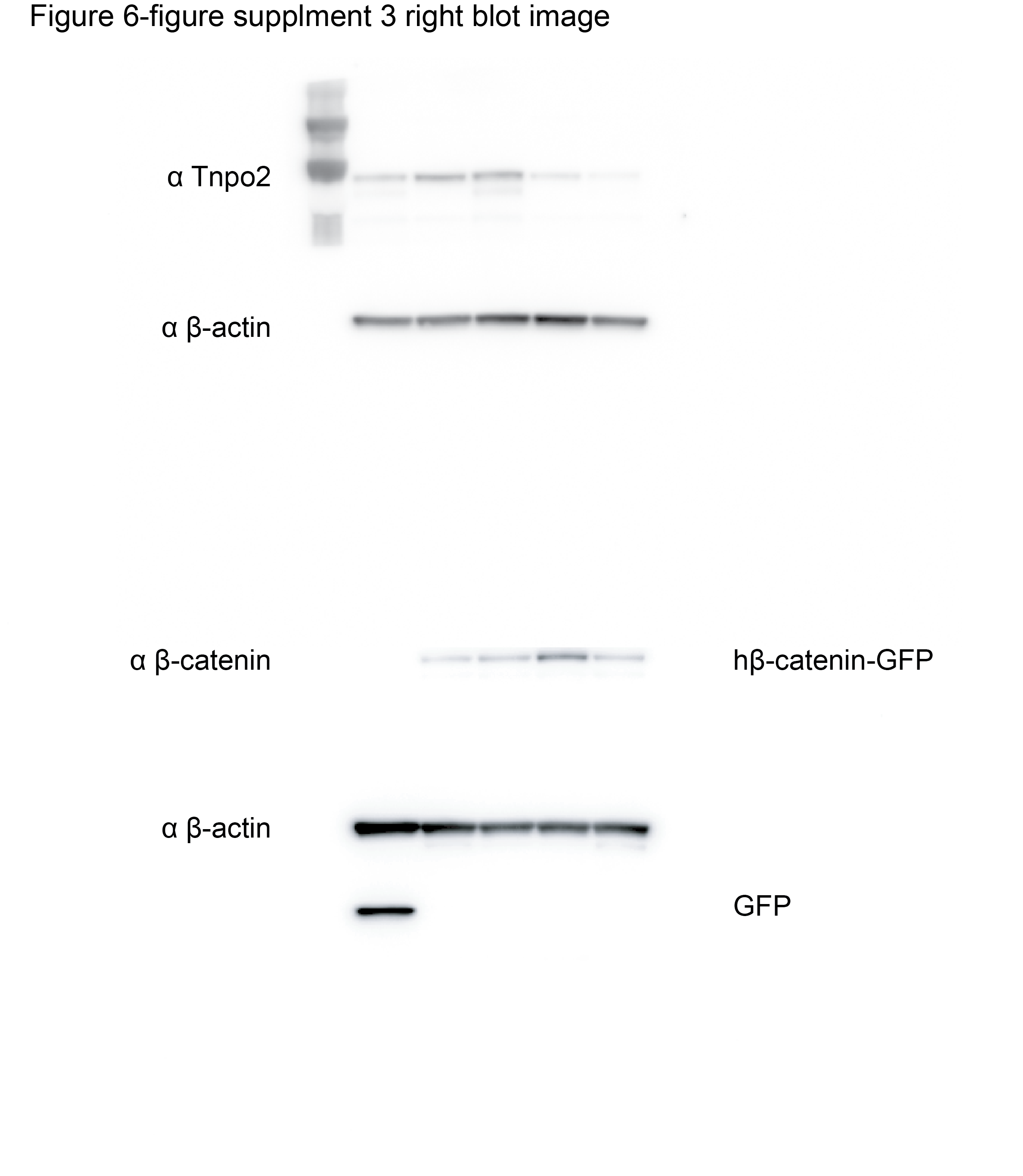

Supplement: Source data 1. [file elife-70495-data1.zip › Source data_gel & blot_revision_10_19_22/Figure 6-figure supplement 3 right blot image.png]

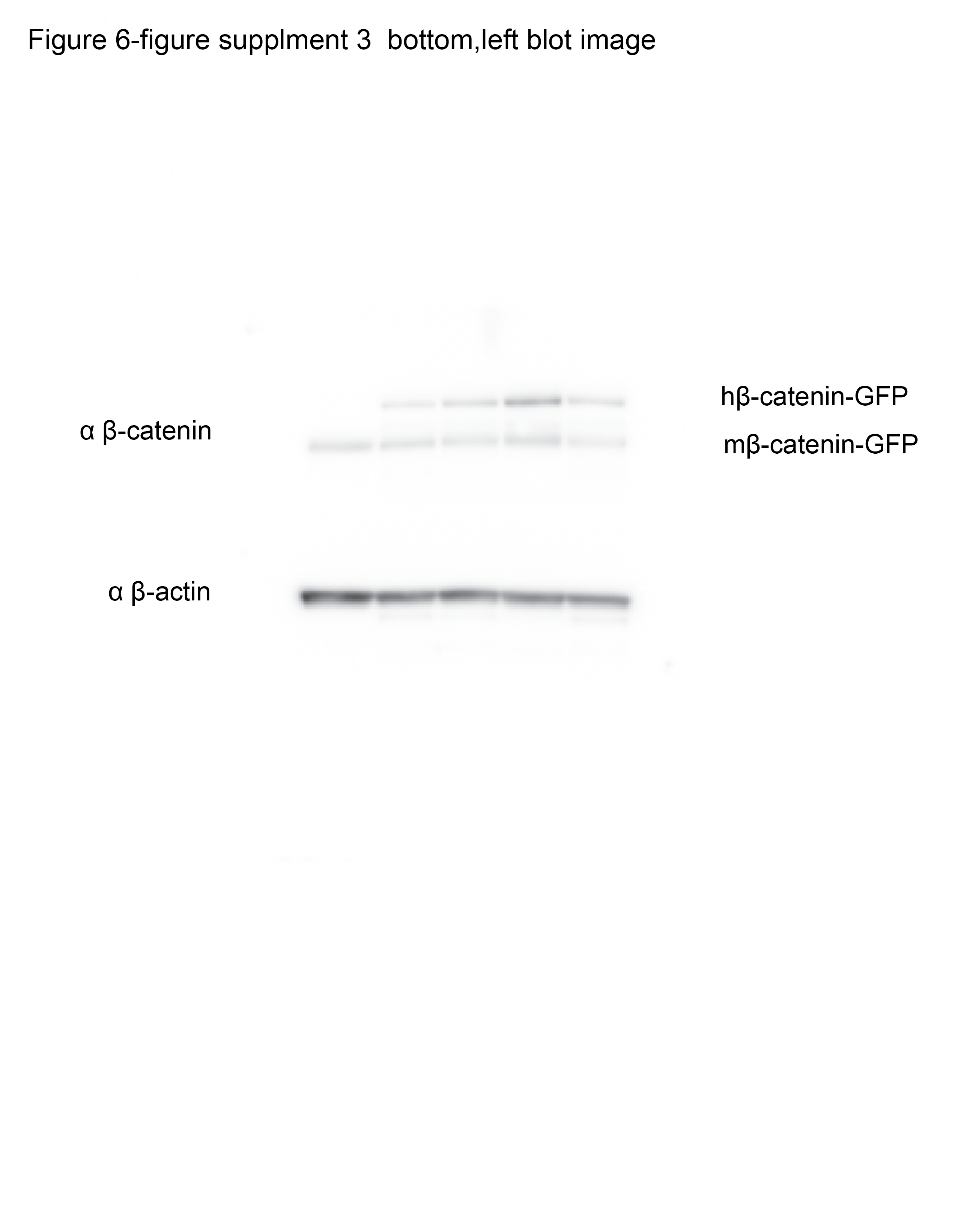

Supplement: Source data 1. [file elife-70495-data1.zip › Source data_gel & blot_revision_10_19_22/Figure 6-figure supplement 3 bottom left blot image.png]

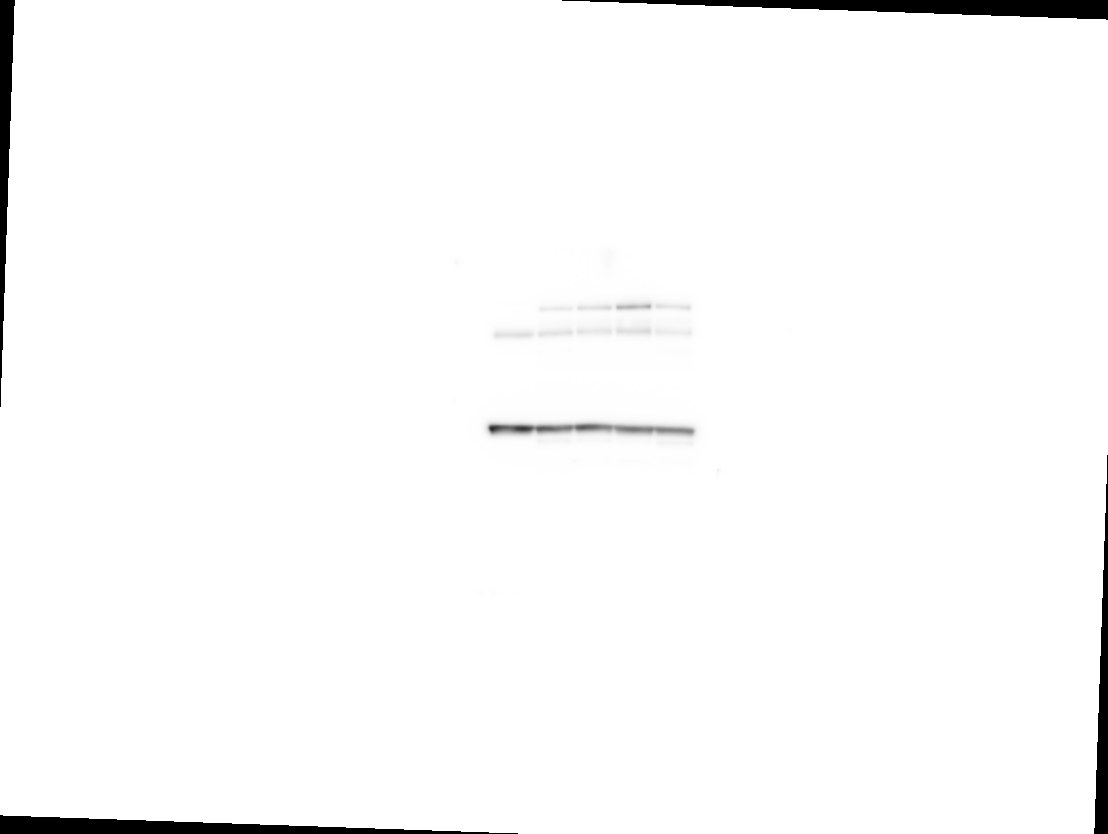

Supplement: Source data 1. [file elife-70495-data1.zip › Source data_gel & blot_revision_10_19_22/Figure 6-figure supplement 3 b-cat and b-actin blot (bottom left)_raw.tif]

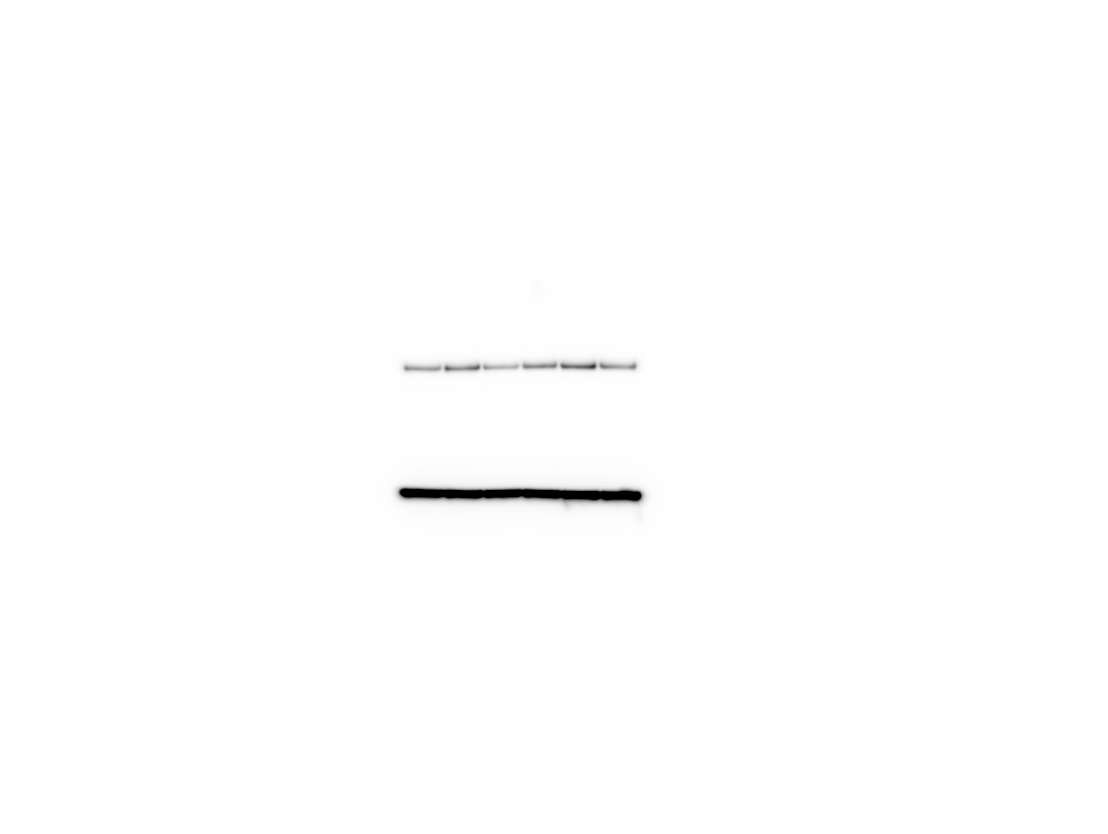

Supplement: Source data 1. [file elife-70495-data1.zip › Source data_gel & blot_revision_10_19_22/Figure 7-figure supplement 1A_raw exposure 2.tif]

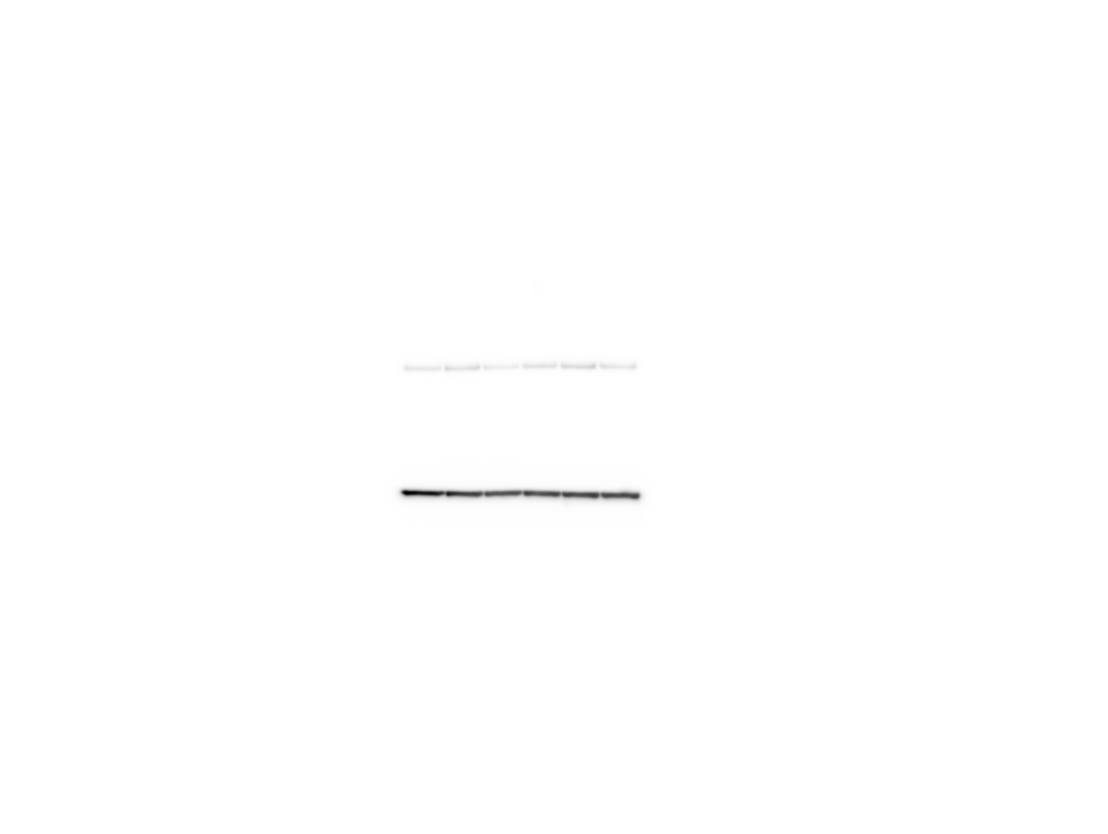

Supplement: Source data 1. [file elife-70495-data1.zip › Source data_gel & blot_revision_10_19_22/Figure 7-figure supplement 1A_raw exposure 1.tif]

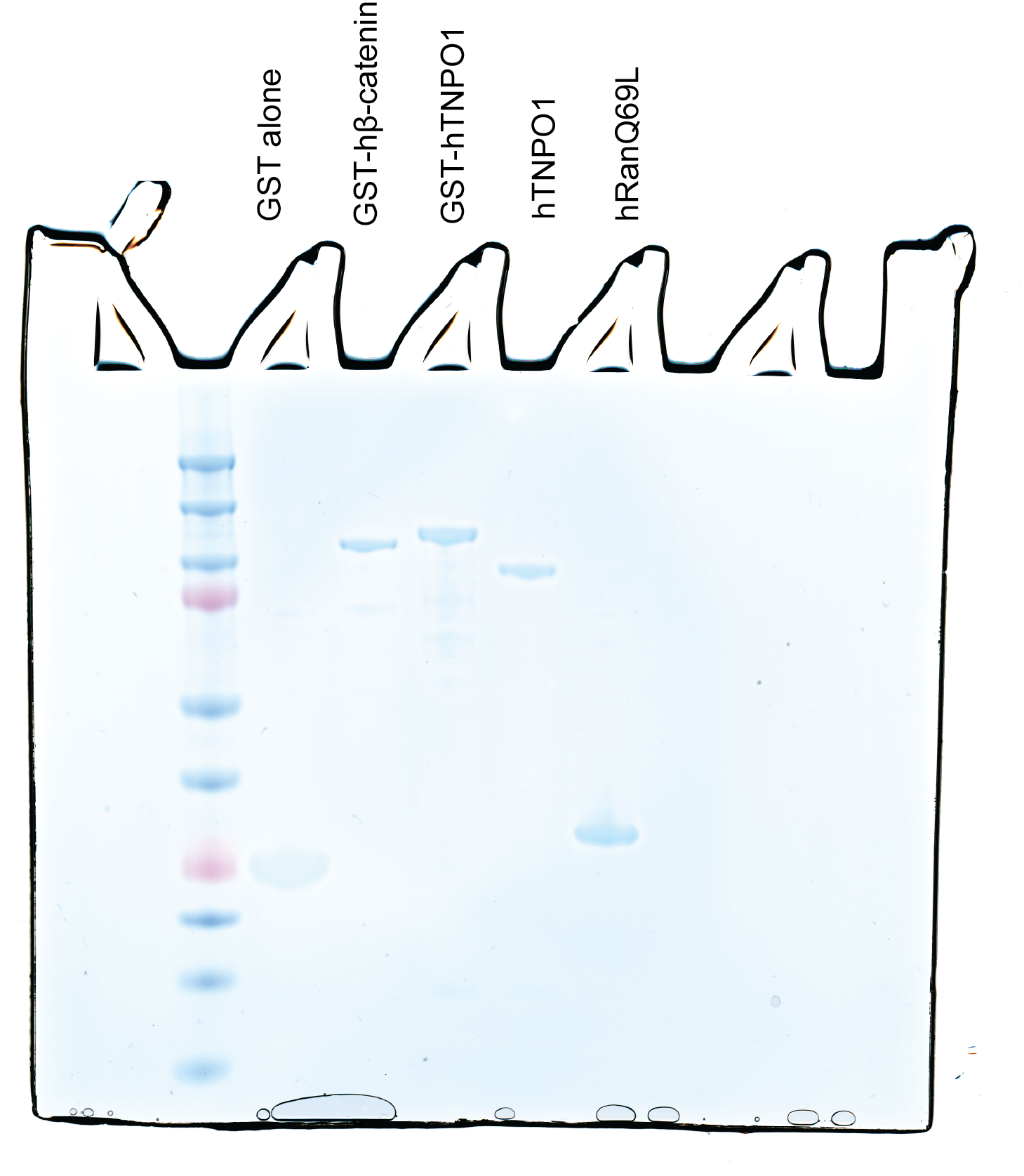

Supplement: Source data 1. [file elife-70495-data1.zip › Source data_gel & blot_revision_10_19_22/Figure 5-figure supplement 1A image.png]

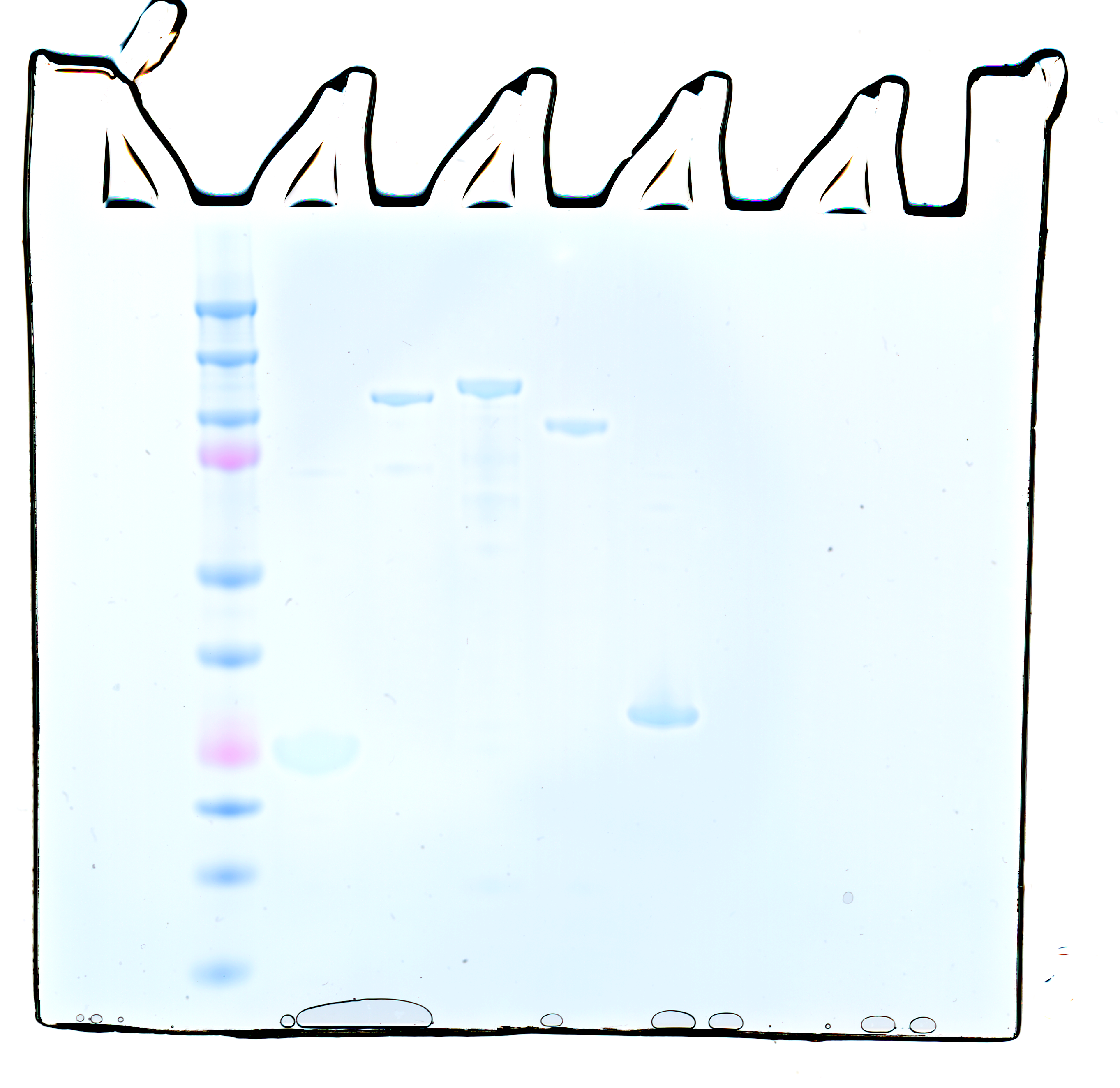

Supplement: Source data 1. [file elife-70495-data1.zip › Source data_gel & blot_revision_10_19_22/Figure 5-figure supplement 1A_raw.tif]

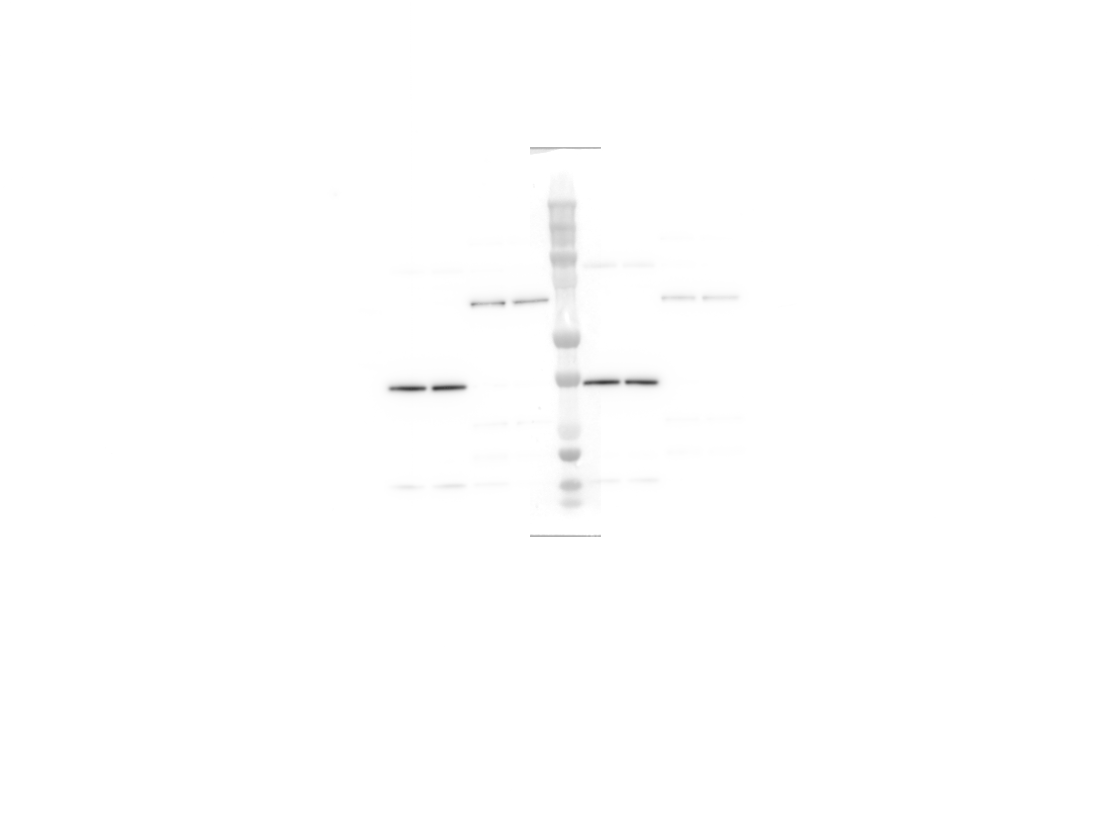

Supplement: Source data 1. [file elife-70495-data1.zip › Source data_gel & blot_revision_10_19_22/Figure 6-figure supplement 4 blot image 1 (bottom)_raw.tif]

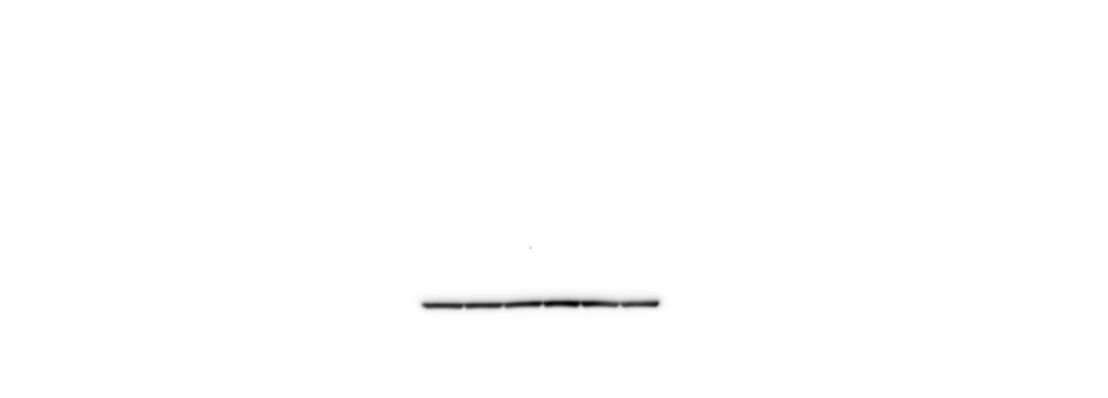

Supplement: Source data 1. [file elife-70495-data1.zip › Source data_gel & blot_revision_10_19_22/Figure 7-figure supplement 1B b-actin ab blot_raw.tif]

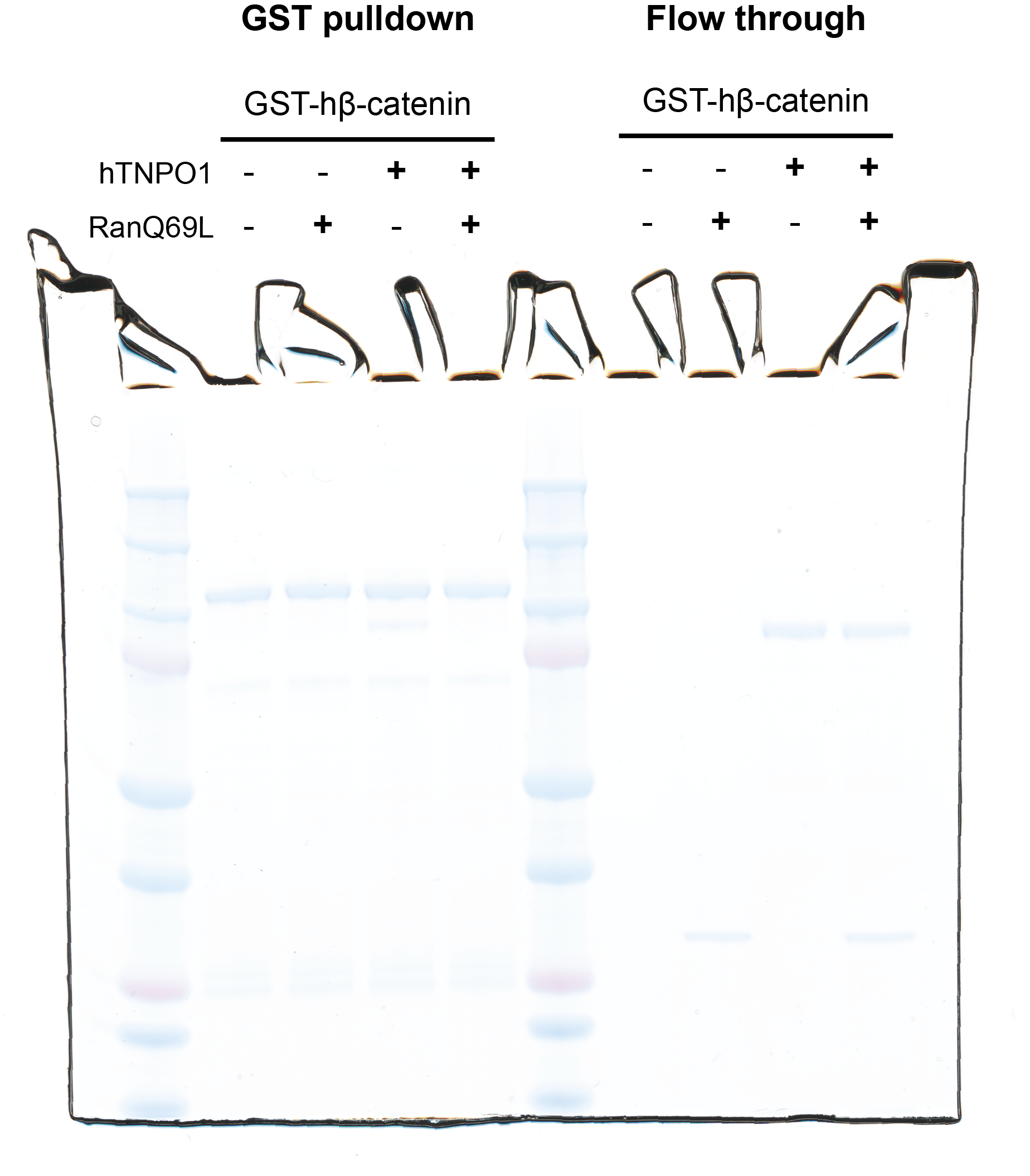

Supplement: Source data 1. [file elife-70495-data1.zip › Source data_gel & blot_revision_10_19_22/Figure 5B image.png]
